# Safety and immunogenicity of single dose live attenuated varicella vaccine (VR 795 Oka strain) in healthy Indian children: A randomized controlled study

**DOI:** 10.1080/21645515.2014.1004031

**Published:** 2015-02-18

**Authors:** Monjori Mitra, Mma Faridi, Apurba Ghosh, Nitin Shah, Raju Shah, Suparna Chaterjee, Manish Narang, Nisha Bhattacharya, Gandhali Bhat, Harish Choudhury, Ganesh Kadhe, Amey Mane, Sucheta Roy

**Affiliations:** 1 Institute of Child Health; Kolkata, India; 2 Department of Pediatrics; University College of Medical Sciences; GTB Hospital Dilshad Garden, Delhi, India; 3 Department of Pediatrics; Lion's Tarachand Bapa Hospital Sion West; Mumbai, India; 4 Ankur Children's Hospital; Ahmedabad, India; 5 Department of Pharmacology; Institute of Postgraduate Medical Education & Research; Kolkata, India; 6 Medical Affairs Wockhardt; East Mumbai, India

**Keywords:** Chicken pox, immunogenicity, live attenuated vaccine, Oka Strain, Varicella vaccine

## Abstract

Varicella, an acute viral systemic infection that may cause lifelong latent infection with the potential for causing clinical reactivation, may be prevented by immunization. The present study was an open label, randomized, controlled, phase III, multicentre trial, conducted to evaluate and compare the safety, tolerability and immunogenicity of a freeze dried live attenuated Oka strain Varicella Vaccine (VR 795 Oka strain) with Varilrix (Oka-RIT strain) in children. A total of 268 healthy Indian children aged 12 months to 12 y with baseline VZV IgG antibody (<100 mIU/ mL) were enrolled, and 256 children completed the study. The extent of rise of VZV IgG antibody titer assessed as 3-fold and 4-fold rise from baseline was found to be significantly higher (89.1% and 85.2%) in the test group as compared to control group (73.4% and 61.7%). The post-vaccination GMT of the test group was significantly higher (112.5 mIU/mL) as compared with the control group (67.8 mIU/mL) (*P* < 0.001). The seroconversion rate considering the 5 gp ELISA units/ml equivalent to 10mIU/ml were similar in the control (96.5%) and the test (98.3%) groups. The adverse events were not different in the control and test groups (*P* > 0.05). The test live attenuated vaccine was found to be highly immunogenic, safe and comparable to Varilrix used in control arm.

## Abbreviations

VZV: Varicella Zoster Virus
AE: Adverse events
SAE: Serious AE
CBC: Complete blood count
PP: Per protocol
GMT: Geometric mean titer
IIT: Intention- to- treat
SST: Serum-separating tube

## Introduction

Varicella infection (chickenpox) is an acute viral systemic^1^ and highly contagious disease caused by Varicella Zoster Virus (VZV), a double-stranded DNA virus of the herpes family.^2^

Varicella has a worldwide distribution, primarily affecting young children,^3-6^ mainly of pre-school age^3,5^ that leads to loss of work days^5,7,8^ and high community cost.^5^ It has a high potential impact on public health in tropical countries. Balraj *et al* reported an overall varicella attack rate of 5.9% in an epidemic investigation of varicella in rural southern India.^9^ An overall seropositivity rate of >70 % (11–15 years) and ∼90% (30 years) was reported in India.^10^

After the natural infection, an individual generally acquires life time immunity, but the virus may reactivate years after to cause herpes zoster (shingles).^11,12^ Though varicella infection can be prevented, modified or treated by VZV immunoglobulin or the antiviral drugs but these are very costly, and mainly applied for postexposure prophylaxis or the treatment of varicella in persons at high risk of severe disease.

The eradication of varicella with universal immunization might be possible, as the only reservoir of virus is human.^2^ At least 90% post exposure protective efficacy is expected when the vaccine is administered within 3 d after exposure to VZV.^13^

Vaccines based on the attenuated Oka-strain of VZV have been proven to be safe and effective in controlling the disease.^14-16^ All live attenuated varicella vaccine provide similar protection against varicella as the VZV strains used in vaccine are derived from same parental Oka virus; however, the degree of viral attenuation and clinical performances may vary. The optimal live attenuated vaccine must show balance between immunogenicity and vaccine related adverse events (AE).^15^ Generally, no adverse reactions are observed after injection of varicella vaccine in children of age 1 to 12 years, but minor local reactions like erythema, swelling, ache, itch, fever etc may appear after injection within 24 hours.^17^

The present study was undertaken to evaluate and compare the safety, tolerability and immunogenicity of a freeze-dried live attenuated (VR 795 Oka strain) varicella vaccine (test vaccine) with the live attenuated Varilrix (Oka-RIT strain) vaccine (control vaccine) in young children. The test vaccine has received regulatory approval by the State Food and Drug Administration, People's Republic of China, 2008 (data on file) but the vaccine has not been evaluated in India.

## Results

### Subjects

**Figure 1.**
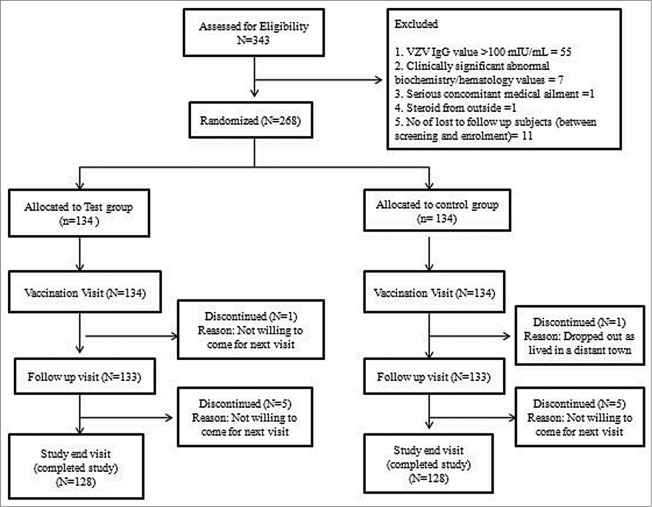
Subject Disposition.

A total of 268 seronegative subjects were enrolled, 12 subjects were lost to follow up during the study and 256 subjects completed the study (**Fig. 1**). The baseline demographic and laboratory parameters in both groups were well-matched (**Table 1***)*. The mean age of the subjects was 46.3   ± 30.3 months and the mean weight was 14.1  ± 6.5 kg.

**Table 1.** Baseline Demographic and Laboratory Parameters of the Enrolled Subjects

Parameters	Total (N = 268 )	Test (N = 134)	Control (N = 134)	P value
Female	110	57	53	0.619
Age (months)*	46.3 ± 30.3	44.5 ± 29.6	48.13 ± 31.1	0.331
12–17	35 (13%)	21 (16%)	14 (10%)	0.282
18–59	160 (60%)	81 (60%)	79(59%)	0.282
60 and over	73 (27%)	32 (24%)	41(31%)	0.282
Weight (Kg)*	14.1 ± 6.5	14.21 ± 7.75	14.1 ± 5.0	0.834
Temperature (°C)	36.7	36.7	36.7	0.519
Pulse Rate (per minute)**	85.7 ± 8.1	85.66 ± 8.12	85.7 ± 8.2	0.946
Hemoglobin (g/dl)*	11.2 ± 1.2	11.2 ± 1.1	11.2 ± 1.3	0.819
TLC (mm^3^)*	10,762.3 ± 3,466.5	11,062.0 ± 3,627.3	10,462.7 ± 3,284.0	0.157
Neutrophils (%) *	42.1 ± 13.0	42.6 ± 13.9	41.61 ± 12.0	0.554
Lymphocytes (%) *	45.1 ± 12.7	44.4 ± 13.1	45.8 ± 12.3	0.410
Eosinophils (%) *	5.4 ± 4.7	5.46 ± 5.1	5.39 ± 4.3	0.902
Basophils (%) *	0.4 ± 0.5	0.39 ± 0.3	0.44 ± 0.6	0.359
Monocytes (%) *	6.4 ± 3.1	6.44 ± 2.8	6.32 ± 3.3	0.760
Platelet Count (lakhs/mm^3^) *	3.2 ± 1 .2	3.3 ± 1 .0	3.2 ± 1.4	0.403

*Mean ± Standard deviation

### Immunogenicity

**Figure 2.**
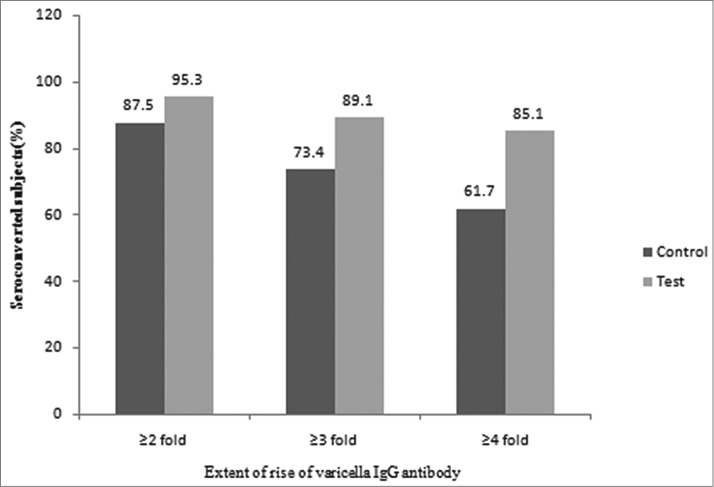
Seroconversion Rate based on Extent of Rise of Varicella (VZV) IgG Antibody from Baseline values to 6 weeks Post Vaccination (N-total no. of evaluated subjects, n-no. of seroconverted subjects).

The seroconversion rate based on extent of rise of VZV IgG antibody titer considering antibody fold rise (2-fold, 3-fold and 4-fold) from baseline values to 6 weeks post vaccination was found to be higher in the test group, (95.3%, 89.1%, 85.1%) as compared with the control group, (87.5%, 73.4%, 61.7%) (**Fig. 2***).*

The seroconversion rate was also analyzed in subjects who had post vaccination titer of VZV IgG antibody titer >10 mIU/mL, which is equivalent to 5gp ELISA units/ml according to the international reference standard.^18,19^ The seroconversion rate showed a similar rate in the control (96.5%) and the test (98.3%) groups.

The GMT (mIU/mL) with 95% CI of 128 subjects in each group and GMT in different age groups (12–17 months, 18–59 months, 60 months and over) are presented in **Table 2**. The post-vaccination increase in GMT from baseline was statistically significant in both the test and control groups (*P <* 0.001, with-in group comparison). The post-vaccination GMT of the test group was significantly higher (112.5 mIU/mL) as compared with the control group (76.8 mIU/mL) (*P <* 0.001, between group comparison).

**Table 2.** GMT of Anti VZV IgG Antibody in Control and Test Group

	Test		Control		
Category	N	GMT mIU/mL (95%CI)	N	GMT mIU/mL (95%CI)	*p*-value
Pre-vaccination	128	13.1 (12.2–14.1)	128	12.6 (11.6–13.6)	<0.001*
12–17 months	21	12 (10.7–13.4)	14	11.3 (10.3–12.4)	0.007*
18–59 months	99	12.6 (11.7–13.6)	89	12.2 (11.3–13.3)	0.001*
>60 months	29	14.9 (12.4–17.9)	39	13.32 (11.2–15.8)	0.066
Post vaccination	128	112.5 (98.3–128.7)	128	67.8 (57.4–80.0)	<0.001*
12–17 months	21	133.3 (93.0–191.1)	14	52.9 (32.0–87.3)	0.007*
12–59 months	99	111.0 (94.9–129.8)	89	64.5 (52.7–79.1)	0.001*
>60 months	29	117.8 (88.50–156.6)	39	75.6 (56.2–101.8)	0.066

*p-value is significant

### Safety

Almost quarter of the subjects in both groups presented pain at the injection site within 48 hours post vaccination. Pain (28.4%), swelling (9%) and redness (3.7%) at the injection site were higher in the test group within 48 hours of post vaccination. After 48 hours post vaccination, 2 patients (1.5%) in the test group and one patient (0.7%) in the control group reported pain at injection site and one patient in the control group reported pain and redness at injection site (0.7%). However, the difference between the control and the test group was not statistically significant (*P >* 0.05) (**Table 3***)* All AE were mild in severity.

**Table 3.** Percentage and Duration of Local AE and Systemic AE within 48 hr Post Vaccination

	Percentage, N (%)			Duration (days) Mean, (min/max)		
Local AE	Test (N = 134 )	Control (N = 134 )	*p*-value	Test (N = 143 )	Control (N = 134 )	*p*-value
Pain	38 (28.4%)	30 (22.4%)	0.260	1 (1,7)	1 (1,3)	0.809
Swelling	12 (9%)	5 (3.7%)	0.078	1 (1,7)	1 (1,1)	1.000
Redness	5 (3.7%)	1 (0.8%)	0.097	1 (1,7)	1 (1,1)	0.799
Bluish discoloration	0	1 (0.8%)	0.315	0	1 (0.1)	NA
**Systemic AE**						
Cough	1 (0.7%)	1 (0.7%)	1.0	1(0,1)	1 (1,5)	0.667
Fever	0	2 (1.5%)	0.154	0	1 (1,3)	NA
Excessive crying	1 (0.75%)	1 (0.75%)	1.000	2 (0,2)	2 (0,2)	1.000

*AE after 48 hr of post vaccination, N-number of subjects, T-no. of local AE in test, c-number of AE in control

Overall, the incidence of systemic AE within 48 hours in both vaccine groups was very low and such AE were mild in nature and lasted for 1–5 d Mild cough and excessive crying were observed in both the groups, but they did not require medication or disturbed the daily activity. None of the patient had fever in the test group while low-grade fever was observed in 2 patients in the control group (**Table 3**).

Systemic AE after 48 hours post vaccination period are presented in **Table 4**. The majority of AE were mild in both the test and the control groups. None of the AE was related to the investigational product. One subject in the control group had an underlying cough for 24 d as the child had a history of wheeze and was on inhaler therapy; hence this was reported as not related to the vaccine by the investigator. Similarly, in the test group one subject had a history of vomiting which developed 20 d after vaccination and was reported as not related to the vaccination by the investigator.

**Table 4.** Percentage and Duration of Local and Systemic AE after 48 hr Post Vaccination

	Test (N = 134) N (%)		Control (N = 134) N (%)	
Adverse event	N (%)	Duration (Days)	N (%)	Duration (days)
Local AE				
Pain Redness	2 (1.49%) 0	1 -	2 (1.5%) 1 (0.8%)	1 1
**Systemic AE**				
Cough	9 (6.7)	3	7 (5.2)	7
Cough and cold	2 (1.5)	4	1 (0.8)	5
Diarrhea	3 (2.24)	4	6 (4.5)	4
Fever	9 (6.7)	0	8 (6.0)	3
LRTI and Fever	1 (0.8)	6	1 (0.8)	4
Otitis externa and fever	1 (0.8)	3	0	0
Maculopapular rash	2 (1.5)	3	1 (0.8)	1
Lymphadenitis	1 (0.8)	8	0	0
Tiredness	1 (0.8)	1	0	1
Generalisedmuscleache	1 (0.8)	3	0	0
Excessive crying	0	0	2 (1.5)	2.5
Scabies	1 (0.8)	2	0	0
Vertigo	1 (0.8)	3	0	0
Vomiting	4 (3.0)	3	2 (1.5)	3
Worm Infection	1 (0.8)	3	0	0
Allergy	0	0	1 (0.8)	6
Vomiting and Headache	0	0	1 (0.8)	1
Insect bite allergy	0	0	1 (0.8)	3

N-total no. of subjects, LRTI: Lower Respiratory Tract Infection in Children

The percentage of overall AE (both local and systemic) during 6 weeks post vaccination were similar in both the test group (49%) and the control group (38%; *p* = 0 .063). Among the test group, 30% of the subjects had local AE (mostly pain, mild in nature), 13% had systemic AE and 6% had both local and systemic AE. In the control group, 20% of the subjects had local AE, 10 % of the subjects had systemic AE and 8% of the subjects had both local and systemic AE. The severity of each AE was evaluated on a 3 point Likert scale (mild = 1, moderate = 2 and severe = 3)

None of the AE was severe in nature or looked varicella-like according to the investigators, thus eliminating the incidence of breakthrough infection.

## Discussion

The results of the present study provide sufficient evidence to prove safety and tolerability of the live attenuated varicella vaccine (VR 795 Oka strain). The test vaccine was immunogenic with acceptable safety and tolerability profile during the entire study. The live attenuated test varicella vaccine developed from Oka strain with mean virus titer of 10^3.4^ was found to be comparable with the control vaccine with 10^3.3^ PFU.^20^

To assess the tolerability and immunogenicity of Oka strain varicella vaccine, various clinical trials have been conducted worldwide which have shown promising results.^21,22^ Oka strain vaccine has been extensively studied in various countries like Japan, United States and several European countries, A good tolerability profile of the vaccine has been reported in healthy as well as immunocompromised children after its use for more than 20 y.^23^ The majority of children are seronegative at an early age of <12 months, hence susceptible to VZV infection. The rate of seroconversion has generally been >95% in healthy subjects after one dose of varicella as reported in various other studies.^24-27^

In India, Ramkisson et al. in their study reported a seroconversion rate of 100% in 176 initially seronegative subjects after vaccine administration.^22^ The protective efficacy of live attenuated varicella vaccine is dependent on vaccine titer. A high-titer varicella vaccine provides high rate of protective immunity and it is well suited for use in healthy young children.^28^ Today, Oka strain containing varicella vaccines are used to immunize approximately 32 million people annually worldwide.^29^

A randomized control trial of the varicella vaccine conducted in China demonstrated good immunogenicity and safely profile of the test vaccine in 291 subjects aged 1–12 y The post vaccination GMT value of 32.8 mIU/mL and a seroconversion rate of 99.3% were reported. No AE were observed during the entire study (data on file). In the present study the rise of antibody with fold3- and fold4- rise post vaccination at 6 weeks was significantly higher in test group than control group. A significant increase in the GMT was observed in the test vaccine compared to the control vaccine that demonstrates good immunogenicity of the test vaccine. The test vaccine is more immunogenic than the control may be due difference in vaccine manufacturing processes.

Our results were consistent with other studies where high GMT was reported after administration of varicella vaccine.^14,22^ Silber et al. reported higher GMT values in subjects of age 12 to 14 months which showed that even the immune system of the young children was able to generate a VZV antibody response.^30^ Kuter et al. considered the subjects of age 13 to 17 months as primary target group in general vaccination program of varicella control.^28^ According to WHO, the optimal age for varicella vaccination is 12–24 months.^13^

In the present study, all of the subjects who received Oka strain varicella vaccine subcutaneously, showed excellent tolerance to vaccine. During the 6 weeks post vaccination follow up period, a very low percentage of unsolicited AE were observed. These results were consistent with the previous literature, where 18% of unsolicited AE was reported after 6 weeks of live attenuated varicella vaccine follow up period.^23^ Most of the local AE in the present study were mild in nature and did not require any medications. Overall, the occurrence of systemic AE within 48 hours in both vaccine groups was very low.

VZV vaccine induces cellular as well as humoral immunity^31^ by eliciting memory T lymphocytes response. Zerboni et al. in their study observed cell mediated immunity to VZV in 85 children and 95 adults immunized with vaccine during 5 y follow up.^32^ Watson et al. reported persistence of cellular and humoral immune responses in a large percentage of vaccinees (91%) for up to 6 y after varicella vaccine immunization, suggesting that the protection against severe varicella is likely to be similarly long -lasting.^33^ Immunity to varicella following vaccination lasts for at least 10–20 y.^13^ More than 90% immunocompetent individuals, previously vaccinated during childhood were still varicella protected, after observing them for 20 y in Japan and 10 y in United States of America.^13^ Further, the oka strain vaccine is genotypically and phenotypicaly stable and do not revert to virulence.^22^ Follow up studies are required to estimate long term persistence of the VZV IgG antibodies following immunization with this vaccine.

In conclusion, the live attenuated varicella test vaccine was found to be highly safe, immunogenic and comparable to the control vaccine, Varilix and can be an option for immunization against VZV in India.

## Methodology

### Study population

A total of 343 healthy Indian children aged 12 months to 12 y of either sex with baseline VZV IgG antibody (<100 mIU/ mL) were screened in the study. The parent(s)/guardian(s) of these children were willing to give written informed consent and comply with all the study related procedures.

The subjects with past history of chickenpox and herpes zoster natural infection, having administered varicella zoster immune globulin or any vaccine/ blood products in the previous 4 weeks, hypersensitivity to any vaccine component, any major congenital abnormality - cardiac, renal, neurological, any acute dermatological disease such as, allergy and bacterial/viral/ fungal infection, participant on any dose of oral/parenteral steroids or inhalational steroids >800 mg of beclomethasone (or its equivalent) in the last 3 months prior to vaccination, febrile (axilliary temperature > 37°C) or any systemic illness at the time of vaccination, any established or clinically suspected immunosuppressive or immunocompromised disorder/state (congenital or acquired- drug induced, neoplastic, tuberculosis etc.) were excluded from the study.

### Analysis of Primary and Secondary Objective

The primary objective of the study was a comparative assessment of the immunogenicity and serocenversion of the test vaccine (VR 795 Varicella Oka strain, manufactured by Changchun Changsheng Life Science Limited, China) versus the comparator Varilix (control vaccine, manufactured by Glaxo Smithkline Beecham Biologicals, UK) by estimation of the extent of rise of VZV IgG antibody from baseline to 6 weeks post vaccination both in terms of geometric mean titer (GMT) and fold4- rise in the antibody titer value.

The secondary objective was the comparative assessment of safety and tolerability of the vaccine up to 6 weeks post vaccination. All AE were observed within 48 hours of vaccination and during the 6 weeks of post vaccination. The severity of each AE was evaluated on a 3 point Likert scale (mild = 1, moderate = 2 and severe = 3) which was obtained from the subject diary given to the parents/guardians of the subjects.

The seroconversion rate in the varicella vaccine doesn't have a WHO recommended cut-off value, all the experiments have to be based on the commercial ELISA kit recommendation which doesn't always have a very realistic results. The commercial ELISAs still lack sufficient sensitivity to reliably detect vaccine seroconversion.^34^ The fluorescent antibody-to-membrane antigen (FAMA) test is generally considered the reference assay for VZV serology^35^ but its use is restricted as it is not amenable to automation. We did not perform FAMA as it was not available commercially. In the present study, we used the antibody fold rise from the baseline to demonstrate the immunogenicity of the vaccine. The subjects were considered seroconverted if there was 4-fold rise of the baseline antibody titer at 6 weeks post vaccination (data on file).

### Study design and procedure

This was an open label, randomized, controlled, phase III, multicentre trial conducted at 4 centers in India (Delhi, Mumbai, Ahmedabad and Kolkata). The study was initiated on November 26, 2012 and completed on June 18, 2013. The study was registered CTRI (www.ctri.nic.in) with the registration number REF/2012/08/003927. The study was conducted in accordance with the principles of the Schedule Y of the Dugs and Cosmetics Rules and Act, ICH and Indian GCP and adhered to the Indian Council for Medical Research guidelines for Biomedical Research on Human subjects (2006) and the declaration of Helsinki.^36^ The investigators had obtained IRB/IEC approval of the protocol, in compliance with local law.

The study consisted of 4 visits. A screening visit (day -7 to -14), vaccination visit (day 0), first follow up visit (day 10) and end of the study visit (6 weeks).At the screening visit, a detailed medical history of the subject was registered. Approximately, 2 mL blood was collected in an EDTA tube and 3.5 mL blood was collected in a serum-separating tube (SST) gel tube and centrifuged for varicella antibody estimation and dispatched on the same day to the central laboratory (Quest Diagnostics, Gurgaon, India). The clinical examination, baseline varicella antibody (IgG) titer estimation and hematological tests (complete blood count, CBC) were performed.

Quantitative antibody titer estimation was done by enzyme immunoassay for IgG antibodies against VZV in serum using the kit VaccZyme, VZV glycoprotein, IgG low level enzyme immunoassay kit MK092 (The Binding Site Ltd, PO Box 11712, Birmingham B14 4ZB, U.K)

At vaccination visit, subjects with baseline seronegative VZV IgG antibody titer were randomized with equal allocation ratio using software generated randomization list. (www.sealedenvelope.com). Allocation concealment was done by serially numbered opaque sealed envelope method. Depending on the randomization list test or control vaccine was administered to each group.

The vaccine in both groups was reconstituted in 0.5 ml of sterile water and was administered subcutaneously in the upper arm or anterolateral thigh of the enrolled subjects. All the vaccinated subjects were observed for any AE immediately and up to 60 minutes post vaccination. A subject diary was also handed over to the parent/ guardian for recording symptoms or signs like pain, swelling or redness at injection site or occurrence of rash, fever etc (up to 10 d post vaccination).

At each follow up visit, subject diary was reviewed for any AE. At the same time medical history was registered and clinical examination was performed. At end of the study visit, symptom diary was again reviewed and safety was evaluated. The medical history of the subject was taken and clinical examination was performed. Blood was collected for antibody estimation and hematological parameters evaluation.

### Statistical Analysis

*Sample Size Estimation:* Two Oka strain vaccine from different manufacturers have shown a difference of ∼10% in response rates in terms of seroconversion published in previous studies.^15^ Assuming this as clinically meaningful difference, the number of subjects per group required to detect a change in seroconversion rates of at least 10%, with an α error 5% and 80% power, was 127. Considering a drop-out rate of 5% the total sample size was estimated to be 268.

The immunogenicity data was analyzed for per protocol (PP) population (vaccinated subjects who attended the 6 week study) and safety data was analyzed for intention- to- treat population (ITT, all the subjects who were randomized).

Continuous variables like age and weight were compared between groups for statistically significant difference if any, using unpaired t-test and categorical variables like sex were compared by Chi-squared test. The pre-vaccination GMT for VZG IgG at baseline was compared with the 6 weeks post vaccination titer for detecting any statistically significant differences, within group by Wilcoxon signed rank test and between groups by Mann-Whitney U test (95% CI, *P* < 0.05).

The occurrence of AE (both serious and non-serious AE) was calculated as the percentage of the vaccinated subjects who developed either local and/or systemic adverse events during the 6 weeks post vaccination period. The safety was compared using Chi-squared tests (95% CI, *P* < 0.05). Data analysis was performed using SPSS statistical software version 15 (IBM SPSS software USA).
